# From freshness to preservation: insights into processing and storage impacts on purple passion fruit volatilome

**DOI:** 10.1002/jsfa.70330

**Published:** 2025-11-20

**Authors:** Alexandre MA Fonseca, Carlos A Pinto, Jorge A Saraiva, Armando JD Silvestre, Sílvia M Rocha

**Affiliations:** ^1^ LAQV‐REQUIMTE and Department of Chemistry University of Aveiro, Campus Universitário de Santiago Aveiro Portugal; ^2^ CICECO and Department of Chemistry University of Aveiro, Campus Universitário de Santiago Aveiro Portugal

**Keywords:** gas chromatography, high‐pressure processing, juice, purple passion fruit, storage, volatilome

## Abstract

**BACKGROUND:**

Purple passion fruit is highly appreciated for its distinctive sweet flavour, low acidity, and aromatic profile, making it a desirable choice for fresh consumption and juice production. To ensure microbiological safety and extend shelf life, juice products typically undergo thermal pasteurization, which can alter their physicochemical properties and volatile composition, and ultimately impact consumer perception. However, the dynamics of the purple passion fruit volatilome, from fresh fruit to processed juice during storage, remain poorly understood. This study aimed to comprehensively characterize these transformations using an advanced analytical technique (two‐dimensional gas chromatography coupled with time‐of‐flight mass spectrometry).

**RESULTS:**

For the first time, the volatile profile of whole and halved fruits was investigated, leading to the identification of 80 previously unreported compounds. Furthermore, high‐pressure processing was compared with thermal pasteurization in terms of its effects on juice quality parameters. The former better retained key attributes of fresh juice after 60 days of refrigerated storage, namely, total phenolic content (76.4% *vs.* 59.6%), antioxidant activity (34.8% *vs.* 2.8%), browning degree (18.7% *vs.* 56.7% increase), colour (Δ*E* = 7.99 *vs.* 15.5), and volatile profile integrity. These results were corroborated by the evaluation of the extension of several reactions (Maillard and Strecker reactions, and carotenoids, lipids and monoterpenic oxidative reactions).

**CONCLUSION:**

Overall, this work provides novel chemical data that may help elucidate consumer perception of distinctive purple passion fruit characteristics at several stages of fruit consumption and provide a scientific basis for the application of high‐pressure processing in producing minimally processed juices with improved preservation of sensory characteristics. © 2025 The Author(s). *Journal of the Science of Food and Agriculture* published by John Wiley & Sons Ltd on behalf of Society of Chemical Industry.

## INTRODUCTION

*Passiflora edulis* f. *edulis* produces highly fragrant passion fruit with a purple peel coloration. Although this variety is less cultivated, it is generally regarded as more palatable and consequently, in addition to its use in juice production, its pulp is also frequently consumed in fresh.^1^ Consumer acceptance of fruits is strongly influenced by attributes shaped during pre‐harvest and post‐harvest stages.^2^, ^3^ Upon purchase, aroma is a key quality indicator, as volatile compounds contribute to flavour, nutritional value, and safety perception.^4^, ^5^ Aroma consists of a unique and complex mixture of volatile organic compounds (VOCs) specific to each fruit species and variety, which is influenced by their combination, concentration, and perception thresholds.^6^

When purchased fresh, the edible part of passion fruit (pulp), responsible for the characteristic passion fruit aroma, is enclosed within the peel (pericarp). This is a rigid spheroid structure, mainly composed of insoluble (cellulose, hemicellulose) and soluble (pectin) fibres that is covered by the cuticle (a lipidic substance composed of cutin and waxes).^1^, ^7^, ^8^, ^9^ At purchase, the perceived aroma reflects the whole fruit volatilome. However, differences in peel composition and its barrier function mean the aroma differs from that perceived at consumption, when the pulp is exposed. Additionally, this exposure triggers enzymatic oxidation, ester formation, and release of bound aromatic compounds.^10^ Passion fruit aroma is often referred as fruity, with a floral, estery and exotic tropical sulfur notes.^11^ Previous studies reported that in passion fruit, as in most fruits, aliphatic esters constitute the predominant class of volatile compounds.^12^, ^13^, ^14^, ^15^ Hexyl hexanoate, ethyl hexanoate, hexyl butanoate, ethyl butanoate, and ethyl acetate are frequently reported among the most abundant compounds.^12^, ^13^, ^14^, ^15^ However, 19 odour‐active compounds were identified as responsible for characteristic odour notes, and most of them are not major components of volatile fraction.^16^ Among these, ethyl butanoate, ethyl hexanoate, and β‐ionone showed the highest odour activity value (OAV) and thus identified as key aroma compounds of this fruit.^16^

Besides direct consumption of the pulp, passion fruit is also frequently processed into juice. In this case, thermal processing technologies such as thermal pasteurization (TP) have been traditionally used to assure juice microbial safety during shelf life. However, high‐temperature processing usually affects the product nutritional and sensorial characteristics, and passion fruit volatile compounds have been shown to be particularly sensitive with up to 35% of its content being lost during thermal treatment.^17^ Besides lowering the amount of volatile compounds, heat may also cause several chemical and nutritional changes in food products, such as browning, colour changes, and formation of undesirable compounds.^18^ High‐pressure processing (HPP) is a nonthermal alternative increasingly used in fruit juice processing for its ability to maintain safety while better preserving nutrients and sensory traits.^19^ HPP, however, may still induce changes such as protein denaturation, enzyme modulation, or alterations in carbohydrates and fats.^20^ In yellow passion fruit purée, HPP preserved colour, sugars, and organic acids better than TP.^21^ Sensory studies also show that HPP‐treated juice and purée retain attributes more similar to fresh juice than thermally processed ones.^21^, ^22^

Although the volatile profile of purple passion fruit has been investigated before, existing literature mainly focus on static snapshots of volatile composition, often limited to a single maturity stage or a specific product form (e.g., juice or pulp). This represents a critical gap, as the volatilome of natural products is known to be highly dynamic and influenced by multiple biotic and abiotic factors, including ripening stage, storage conditions, and processing methods. Thus, a comprehensive approach to purple passion fruit volatile dynamics throughout postharvest handling of the fruit and juice processing and storage, remains largely underexplored. This approach could provide valuable insights into the modifications/reactions throughout the fruit value chain with impact on physicochemical and sensorial characteristics. Understanding these dynamics is essential for optimizing postharvest practices, enhance flavour quality, and develop products that retain the characteristic sensory attributes of the fresh fruit with clear benefits for the final consumer. Hence, our study relied on the use of an advanced analytical technique – two‐dimensional gas chromatography coupled with time‐of‐flight mass spectrometry (GC × GC‐ToFMS) – to: (i) characterize for the first time the volatilome of whole (WF) and halved (HF) purple passion fruit and identify compounds that potentially impact consumer aroma perception; (ii) characterize the fresh juice volatilome and investigate the impact of HPP and TP on its aroma profile and physicochemical characteristics to assess the methodology that better preserves the characteristics of the fresh juice; and (c) track the evolution of key aroma compounds and processing markers of juice processing over a 60 days storage.

## MATERIALS AND METHODS

### Chemicals

Gallic acid monohydrate (>99.5%) and l(+)‐ascorbic acid (>99,7%) were supplied by Scharlab SL (Barcelona, Spain). Folin–Ciocâlteu reagent was purchased from Biochem Chemopharma (Cosne‐Cours‐sur‐Loire, France). Methanol (>99.8%) and potassium persulfate (>99.0%) were purchased from Chem‐Lab NV (Zedelgem, Belgium). 2,2‐Diphenyl‐1‐picrylhydrazyl hydrate (DPPH) and 6‐hydroxy‐2,5,7,8‐tetramethylchroman‐2‐carboxylic acid (97%) (Trolox) were supplied by Sigma Chemical Co. (Madrid, Spain). 2,2‐Azinobis‐3‐ethylbenzothiazoline‐6‐sulfonic acid (ABTS) (98%) was supplied by Thermo Fisher GmbH (Kandel, Germany). Sodium carbonate (>99.8%) was purchased from Scharlab SL. Sodium chloride (≥99%) was purchased from Honeywell Specialty Chemicals Seelze GmbH (Seelze, Germany).

### Sampling

Fruits (5 kg) were harvested from an irrigated purple passion fruit (*P. edulis* f. *edulis*) organic orchard located in Paredes de Viadores, Portugal (41° 07′ 03.2″ N; 8° 06′ 23.6″ W). Water was supplied through a drip irrigation system from May until October every 2 days to avoid water stress on plants. Fruits used in this study were collected in the last 2 weeks of September in fully ripe state (full and homogeneous purple pigmentation of the peel and before falling from the plant). Sampled fruits were immediately transported to the laboratory, washed with tap water, the peduncle was removed and fruits were left to dry at room temperature. Fruits were subsequently used for WF and HF volatile profile determination. In parallel, juice was obtained by halving the fruits and separating the pulp from the peel with a spoon. The pulp was filtrated with a sterile gauze to remove solid particles, and fresh juice (FJ) was immediately subjected to volatile profile determination and physicochemical characterization or pasteurized and subsequently subjected to the same analysis.

### Juice pasteurization processing and storage

Three independent freshly made juice batches (*n* = 3 for each treatment) were prepared and 25 mL samples were pasteurized either by TP or HPP in heat‐sealed multilayer polyamide/polyethylene bags.

#### Thermal pasteurization

Three juice batches (*n* = 3) were thermally pasteurized according to previously reported conditions.^23^ Briefly, TP was carried out by submerging the juice samples in a thermostatic bath (Selecta Frigiterm 6000382, Barcelona, Spain) at 88.0 °C. The sample temperature was monitored with a K‐type thermocouple (Thermometer 305, Roline, Bassersdorf, Switzerland) connected to a digital thermometer. As soon as the desired temperature was reached, the samples were held in the water bath for 15 s and subsequently cooled in ice. The average come‐up time was 4:27 min. Subsequently, samples were stored at 4 °C up to 60 days and evaluated along this period for their physicochemical parameters and volatile compounds composition.

#### High‐pressure pasteurization

Three juice batches (*n* = 3) were high‐pressure pasteurized using a 55 L capacity industrial‐scale apparatus (model 55, Hiperbaric, Burgos, Spain), under two different conditions: 450 MPa for 10 min (*n* = 3) or 550 MPa for 5 min (*n* = 3) at room temperature (≈22 °C). Tap water was used as pressurization fluid and pressure was generated in about 60 s, whereas pressure release time was less than 3 s. After processing, the samples were immediately cooled in an ice bath and stored at 4 °C for up to 60 days, and evaluated during this period for their physicochemical parameters and volatile compounds composition.

### Juice physicochemical characterization

Freshly made and pasteurized juices were chracterized by several physicochemical parameters during 60 days of storage – *t* = 0 (just after pasteurization), 7, 15, 30, 45 and 60 days – at 4 °C to study the impact of the pasteurization methods and their variation during the shelf life of the passion fruit juice.

#### pH, total soluble solids, and titratable acidity

The pH of juice samples was measured at room temperature with a properly calibrated glass electrode by submerging it into the homogenized samples (micropH 2002, Crison Instruments, SA, Barcelona, Spain).

Total soluble solids content (TSS) was established through °Brix measurement using a digital refractometer (HI96813, Hanna Instruments, Woonsocket, RI, USA).

Titratable acidity (TA) was measured according to AOAC indicator method 942.15 using 0.10 mol L^−1^ NaOH (Panreac, Barcelona, Spain) and phenolphthalein as indicator.^24^ Results are expressed as grams of citric acid per 100 mL (g CA 100 mL^−1^). All measurements of the mentioned parameters were performed in triplicate for each sample.

#### Colour analysis

The colour of the juices was determined using a spectrophotometer (CM 2300d, Konica Minolta, Tokyo, Japan). Colour parameters – L*, lightness (0, dark; 100, light); a*, redness (+, red; −, green); and b*, yellowness (+, yellow; −, blue) – were obtained at room temperature using the CIELAB system. The CIELAB parameters were determined using the original SpectraMagic NX Software (Konica Minolta) in accordance with the regulations of the International Commission on Illumination. Total colour difference (Δ*E*) was calculated using the following Eqn (1):

(1) $$∆E=\sqrt{{\left(L^{*}-L_{0}^{*}\right)}^{2}+{\left(a^{*}-a_{0}^{*}\right)}^{2}+{\left(b^{*}-b_{0}^{*}\right)}^{2}}$$

where L*, a*, and b* correspond to the measured brightness, redness, and yellowness values of each treated juice, and the coordinates with the subscript ‘0’ are the coordinate values of fresh juice.

#### Browning degree

Browning degree (BD) determination was adapted from a previous study.^25^ Briefly, juice samples were centrifuged at 10 000 × *g*, 4 °C for 10 min and the absorbance of the supernatant was measured at 420 nm in an ultraviolet–visible microplate spectrophotometer (Multiskan GOThermo, Fisher Scientific, Waltham, MA, USA). BD was determined as 10 times the measured absorbance, BD = *A*_420 nm_ × 10.

#### Total phenolic content

Total phenolic content (TPC) was determined via the Folin–Ciocalteu assay, using a method adapted from a previous study.^26^ Briefly, the juice samples were diluted eight times with water and 30 μL of the diluted sample was placed in selected wells of the 96‐well plate. For the blank, 30 μL distilled water was used instead of the sample. For the calibration curve, 30 μL gallic acid standard solutions (7.0–220 mg L^−1^) were placed in the selected wells of the 96‐well plate. Then, 150 μL water‐diluted Folin–Ciocâlteu reagent (1:10) and 120 μL Na_2_CO_3_ (75 g L^−1^) solution were added to each well. After 5 min incubation in the dark at 50 °C, the absorbance of each well was determined at 760 nm using an Eon microplate reader (BioTek Instruments, Inc., Winoosky, VT, USA). Measurements were performed in three replicates of each sample and their average and standard deviation expressed as milligrams of gallic acid equivalents per 100 mL of juice (mg GAE 100 mL^−1^).

#### Antioxidant activity

DPPH antioxidant activity determination was adapted from a previous study.^26^ Briefly, the juice samples were diluted eight times with water and 75 μL of the diluted sample was placed in the selected wells in the 96‐well plate. For the blank, 75 μL of water was used, and for the calibration curve, 75 μL of Trolox standard solutions (2–35 mg L^−1^) was placed in the selected wells of the 96‐well plate. Then, 30 μL DPPH (125 mmol L^−1^ in MeOH) and 195 μL MeOH were added to each well. After 30 min incubation in the dark at room temperature, the absorbance of each well was determined at 517 nm using an Eon microplate reader (BioTek Instruments). Measurements were performed in three replicates of each sample and their average and standard deviation were expressed as milligrams of Trolox equivalents per 100 mL of juice (mg TE 100 mL^−1^).

#### Statistical analysis

The results obtained for the above‐mentioned parameters were subjected to statistical analysis to determine significant differences between juice treatments (at *t* = 0) and within each juice treatment across storage time. Normality of the data was assessed using the Shapiro–Wilk test, while homogeneity of variances was evaluated using the Brown–Forsythe test. Both assumptions were met (*P* > 0.05) and, therefore, two‐way analysis of variance (ANOVA) was performed, followed by Tukey's test (*P* < 0.05) using Prism, version 9 (GraphPad Software Inc., San Diego, CA, USA).

### Volatile profile determination: fruit and juice

**Figure 1:**
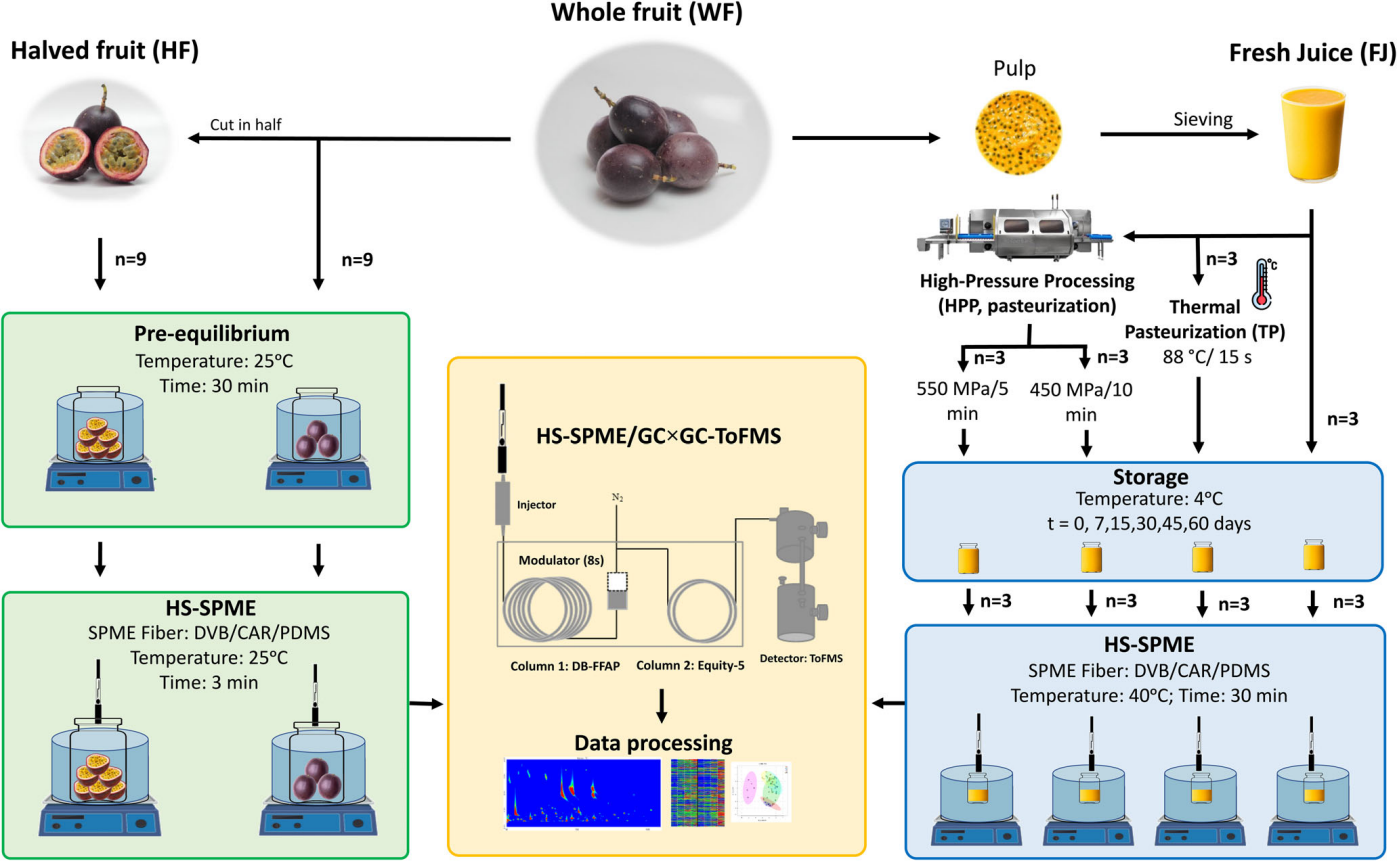
Workflow for determining the volatile profile of whole fruit (WF), halved fruit (HF), and juice (fresh, HPP, or TP pasteurized). WF and HF samples (*n* = 9) underwent short headspace solid‐phase microextraction (HS‐SPME) extraction to simulate consumer aroma perception and were analysed by comprehensive two‐dimensional gas chromatography coupled with time‐of‐flight mass spectrometry (GC × GC‐ToFMS). In parallel, fresh (FJ) and pasteurized independent juices (*n* = 3) were analysed by HS‐SPME (longer extraction at higher temperature) and GC × GC‐ToFMS over storage (0–60 days).

Headspace solid‐phase microextraction followed by comprehensive two‐dimensional gas chromatography coupled with time‐of‐flight mass spectrometry (HS‐SPME/GC × GC‐ToFMS) was employed to the in‐depth study of WF, HF, and juice (FJ, TP, and HPP) volatile profile as illustrated by the workflow in Fig. 1. Sampling, reporting of chemical analysis and data pre‐processing, pre‐treatment, processing, and interpretation were performed according to the metabolomics standards initiative (MSI), as described below.^27^

#### HS‐SPME

Different HS‐SPME conditions were used according to the sample and analysis purpose:

- *Whole and halved fruit*. To replicate the conditions under which consumers perceive the fruit's aroma during purchase and consumption, the volatile profile of passion fruit was analysed in two different forms: WF and HF. For this purpose, a methodology adapted from a previous study was used.^28^ Each analysis comprised the random selection of three fruits that were used directly or previously cut in half before being sealing in a 1 L airtight jar, fitted with a rubber septum in the lid. Jars were closed and kept at room temperature for 30 min during a pre‐equilibrium phase. Then, each jar was immersed in a water bath adjusted to 25.0 ± 0.1 °C, and the DVB/CAR/PDMS SPME fibre was inserted in the headspace through the septum. A very short SPME extraction time (3 min) was used to replicate the conditions under which consumers perceive the fruit's aroma during purchase and consumption.
- *Juice*. For the analysis of passion fruit juices, a methodology adapted from a previous study was used.^14^ Briefly, a twofold water‐diluted fruit juice sample (2.7 mL) was placed in a 13 mL glass vial along with NaCl (10% w/w) and a stirring bar (12 mm × 3 mm). Then, the vial was capped and placed in a thermostatic bath adjusted to 40.0 ± 0.1 °C. The DVB/CAR/PDMS SPME fibre was inserted in the headspace through the septum for 30 min.

#### GC × GC‐ToFMS

The volatiles adsorbed and absorbed on the SPME fibre coating were determined using a LECO Pegasus 4D GC × GC–ToFMS system (LECO, St Joseph, MI, USA) consisting of an Agilent GC 7890A gas chromatograph (Agilent Technologies, Inc., Wilmington, DE, USA), with a dual‐stage jet cryogenic modulator (licensed from Zoex) and a secondary oven, and a mass spectrometer equipped with a ToF analyser. After the extraction/concentration step, the SPME fibre was manually introduced into the port at 250 °C for 3 min for analyte desorption. The injection port was lined with a 0.75 mm ID glass liner. Splitless conditions (30 s) were used. A DB‐FFAP column (30 m × 0.25 mm I.D., 0.25 μm film thickness; J&W Scientific Inc., Folsom, CA, USA) was used as the first‐dimension column (1D) and an Equity‐5 column (0.79 m × 0.25 mm ID, 0.25 μm film thickness; Supelco, Bellefonte, PA, USA) was used as the second‐dimension column (2D). The carrier gas was helium at a constant flow rate of 2.50 mL min^−1^. The primary oven temperature was programmed from 40 °C (1 min) to 140 °C at 2 °C min^−1^, followed by a second ramp from 140 to 240 °C (2 min) at 15 °C min^−1^. Secondary oven program was 5 °C offset above the primary one. Both the MS transfer line and MS source temperatures were set at 250 °C. The modulation period was 8 s, keeping the modulator at 15 °C offset above the primary oven, with hot and cold pulses of 0.90 and 3.10 s, respectively. The mass spectrometer ran in electron ionization mode at 70 eV, using an *m*/*z* range of 30–300. Total ion chromatograms were processed using the automated data processing software ChromaTOF (LECO) at signal‐to‐noise threshold of 100. Spectral deconvolution was computationally processed, being intended to reconstruct clean mass spectra for each component; while the GC × GC peak area was obtained by transforming the series of side‐by‐side second‐dimension chromatograms into a two‐dimensional chromatogram, the GC peak area being proportional to the generated signal intensity.^29^ Contour plots were used to evaluate the general separation quality and for manual peak identification. For identification purposes, the mass spectrum of each detected metabolite was compared with mass spectral libraries, namely an in‐house library of standards and two commercial databases (Wiley 275 and US National Institute of Science and Technology (NIST) V. 2.0 – Mainlib and Replib). A mass spectral match factor, similarity >700/1000, was used to decide whether a peak was correctly identified. Moreover, a manual analysis of mass spectra was performed, combining additional information like linear retention index (RI) value, which was experimentally determined according to the van den Dool and Kratz RI equation.^30^ A C8–C20 *n*‐alkane series was used for RI determination, comparing these values with those reported in the literature for chromatographic columns similar to the above‐mentioned 1D column. A 5% difference limit was used when comparing the calculated linear retention index (RI_calc_) with literature data (RI_lit_).

#### Data processing

A full data matrix consisting of 182 variables (metabolites) and 54 observations was constructed (Supporting Information, Table S1). The 54 observations correspond to WF, HF, FJ, thermically pasteurized juice (TP), high‐pressure pasteurized juice at 450 MPa for 10 min (HPP450), and high‐pressure pasteurized juice at 550 MPa for 5 min (HPP550). Nine samples were analysed in the case of WF and HF and three independent replicates were analysed in triplicate for juice samples. Using MetaboAnalyst 6.0 (web interface) software, autoscaling normalization of the data was applied and heatmap visualization was obtained on this matrix, using absolute GC peak area. Additionally, hierarchical clustering analysis (HCA) was performed using the same software, to further examine the differences and similarities between each condition metabolite profiles. Ward's minimum variance algorithm method and squared Euclidean distances were employed.

## RESULTS AND DISCUSSION

### Volatile dynamics of fresh purple passion fruit

Representative three‐dimensional (3D) GC × GC‐ToFMS total ion chromatogram contour plots of WF and HF are illustrated in Supporting Information, Fig. 2(A),(B), respectively. Here, the headspace metabolite separation is shown according to physicochemical characteristics, namely, through polarity (first dimension) and volatility (second dimension), when a polar and non‐polar set of columns is used. Thus, a structured chromatogram is obtained, in which structurally related analytes occupy similar two‐dimensional (2D) spaces. Each sample produced a chromatogram with approximately 380 (WF) and 590 (HF) instrumental features, and a targeted analysis focused on chemical families such as alcohols, aldehydes, esters, ketones, and monoterpenes was performed. In total, 102 (WF) and 134 (HF) compounds were putatively identified and selected for further analysis. Supporting Information Table S1 lists all selected compounds with chromatographic data and peak areas, including 80 that have not been previously reported in purple passion fruit.

**Figure 2:**
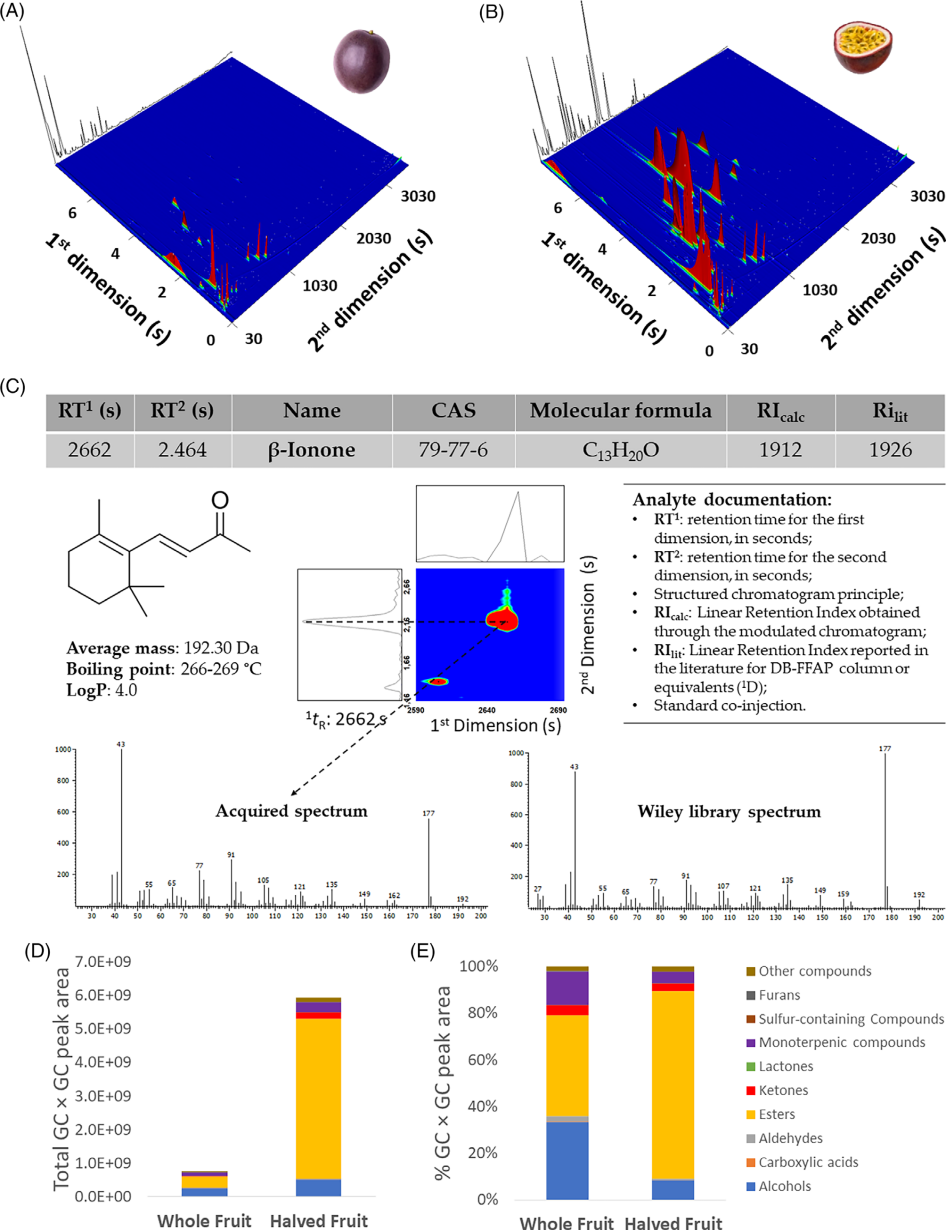
Volatile profile characterization of whole and halved purple passion fruit by GC × GC‐ToFMS. GC × GC‐ToFMS 3D chromatogram plots of (A) whole and (B) halved purple passion fruit released volatile compounds. (C) Example of metabolite documentation procedure to putatively identify β‐ionone (a key aroma compound of purple passion fruit). (D) Contribution of each chemical family to the total chromatographical area and (E) chemical family area percentage of the identified volatile compounds (Supporting Information, Table S1) in whole (WF) and halved (HF) purple passion fruit.

The identification of these analytes requires a comprehensive evaluation of several experimental parameters obtained from the GC × GC analysis, data extracted from databases, as well as physicochemical properties of the molecules. As an example, the metabolite documentation for β‐ionone, a compound of interest in purple passion fruit aroma, is presented in Fig. 2(C) showing its chromatographic data, including RI_calc_ and acquired mass spectrum, with the respective comparative data from the literature and libraries that were used for metabolite identification. This approach, although time‐consuming, increases the confidence in analyte identification.

The contribution of each chemical family to the total chromatographical area (Fig. 2(D)) and their respective percentages (Fig. 2(E)) in WF and HF samples are also presented. In accordance with the differences seen in chromatograms, Fig. 2(D) also shows that HF has a significantly higher amount of released volatile compounds: nearly eight times more total chromatographic area in HF than WF in the dataset of the identified compounds. This increase of released volatile compounds in HF is due to an additive effect of compounds released from the exposure of the fruit pulp as well as resulting from several enzymatic reactions promoted by the opening of the fruit (which disrupts the fruit tissues). As previous studies have shown, pulp volatile compound composition is significantly different from the peel,^10^ and aliphatic esters constitute the predominant class of volatile compounds in purple passion fruit pulp (hexyl hexanoate, ethyl hexanoate, hexyl butanoate, ethyl butanoate and ethyl acetate are frequently reported among the most abundant).^12^, ^13^, ^14^, ^15^ In accordance, a substantial increase in esters' peak area percentage in HF (80.2% and 43.3% in HF and WF) is observed, and a consequential reduction in other families' percentage of total area, despite an increase in their absolute area values.

**Figure 3:**
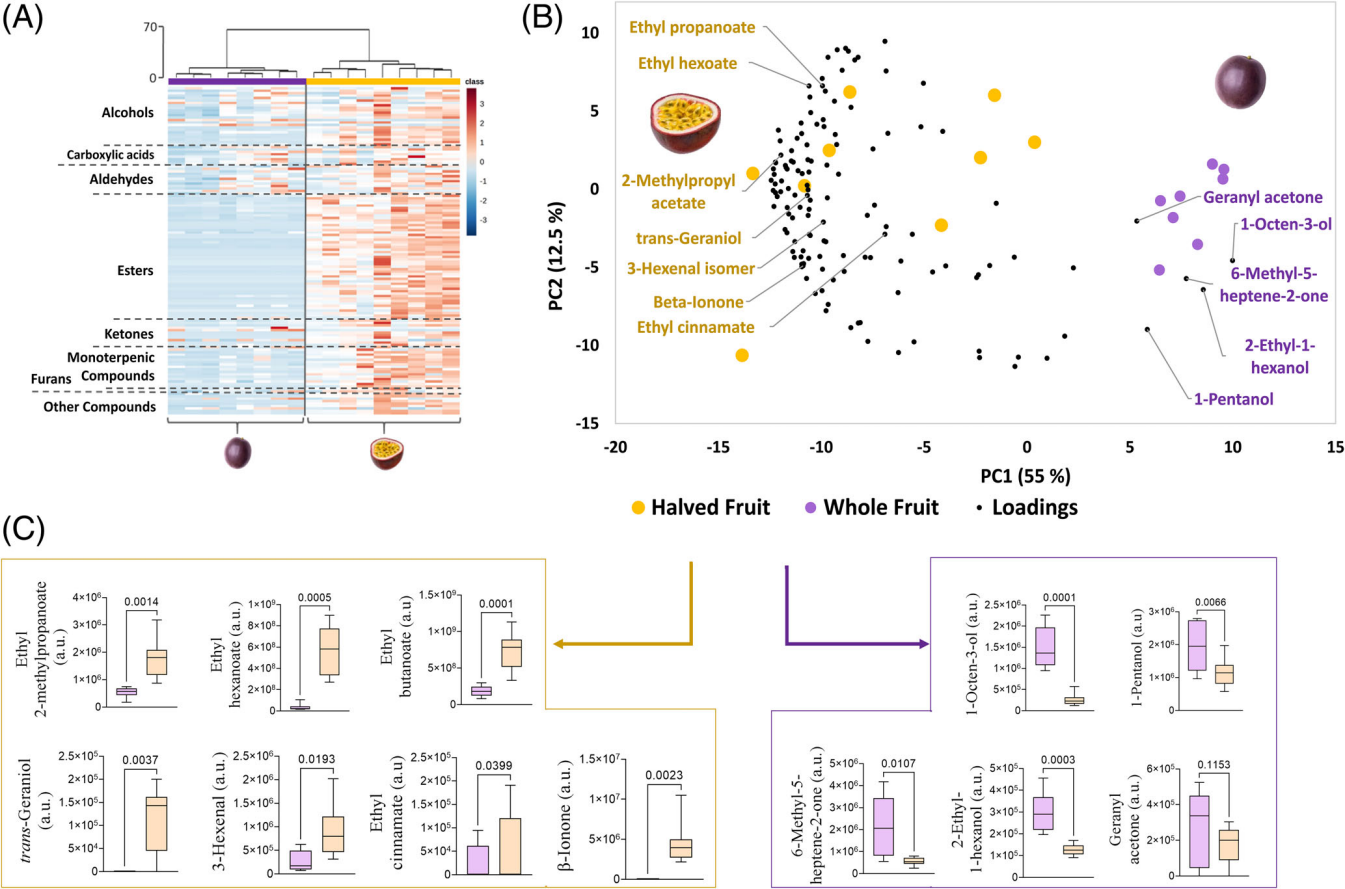
Volatile compound differentiation between whole and halved purple passion fruit. (A) Hierarchical clustering analysis of identified volatile compounds in whole and halved fruit. Heatmap visualization shows the relative content of each compound, through a chromatic scale (from low (blue) to high chromatographic area (red)), which corresponds to its peak area normalized by autoscaling. (B) Biplot representation of observation scores and variable loadings within the principal component space. Compounds highlighted in yellow correspond to the aroma‐active volatile compound reported for purple passion fruit pulp, while highlighted in purple are the compounds that mostly contribute to the distinction of WF. (C) Boxplot representation of all highlighted compounds; a.u., arbitrary units.

Although volatile composition of passion fruit pulp and juice has been explored before, and as far as the authors are aware, this is the first time that the volatile profile of purple passion fruit as a whole, and opened in half, is reported. As such, to further investigate what characterizes the volatilome of these samples, a multivariate analysis was performed on the complete dataset of identified compounds (Supporting Information, Table S1). Figure 3(A) contains a heatmap representation of the GC × GC peak areas for each sample that shows a clear separation of WF and HF samples into two clusters organized into two main branches of the dendrogram, separated by a Euclidean distance of 70. Greater variability within HF is also observed through a higher distance within this group's samples. The rows of the heatmap represent each compound organized into chemical families, which allows the observation that the greatest difference in terms of relative content is clearer in esters and monoterpenic compounds, which corroborates the data observed in Fig. 2(D),(E).

The biplot (Fig. 3(B)) from the principal component analysis (PCA) integrates observation scores and variable loadings within the principal component space. This representation reveals that the reported aroma‐active volatile compounds are correlated and contribute to the differentiation of HF samples, suggesting a lower release of these compounds in WF. Their relative content and statistical differences between samples are illustrated in Fig. 3(C). These compounds are primarily associated with fruity (ethyl 2‐methylpropanoate, ethyl hexanoate, ethyl butanoate), floral (*trans*‐geraniol, β‐ionone), and balsamic (ethyl cinnamate) odour descriptors. Notably, among the three key aroma compounds identified in the literature (ethyl butanoate, ethyl hexanoate, and β‐ionone), only ethyl butanoate and ethyl hexanoate were detected in WF, although at significantly lower levels (4 and 17 times less, respectively). On the other hand, this analysis also highlights the compounds that primarily contribute to the distinction between WF. These include alcohols (1‐octen‐3‐ol, 2‐ethyl‐1‐hexanol, and 1‐pentanol), which exhibit earthy, citrus, and fermented odour notes, as well as ketones (6‐methyl‐5‐hepten‐2‐one and geranyl acetone) associated with a citric aroma.

### Impact of thermal and high‐pressure pasteurization on juice physicochemical characteristics and volatile compound composition

The impact of TP and HPP pasteurization on purple passion fruit juice was evaluated, aiming to provide insights into the changes that each processing method imparts on physicochemical parameters and volatile compound profile of the juice and how these evolve during storage.

#### Physicochemical characteristics

**Figure 4:**
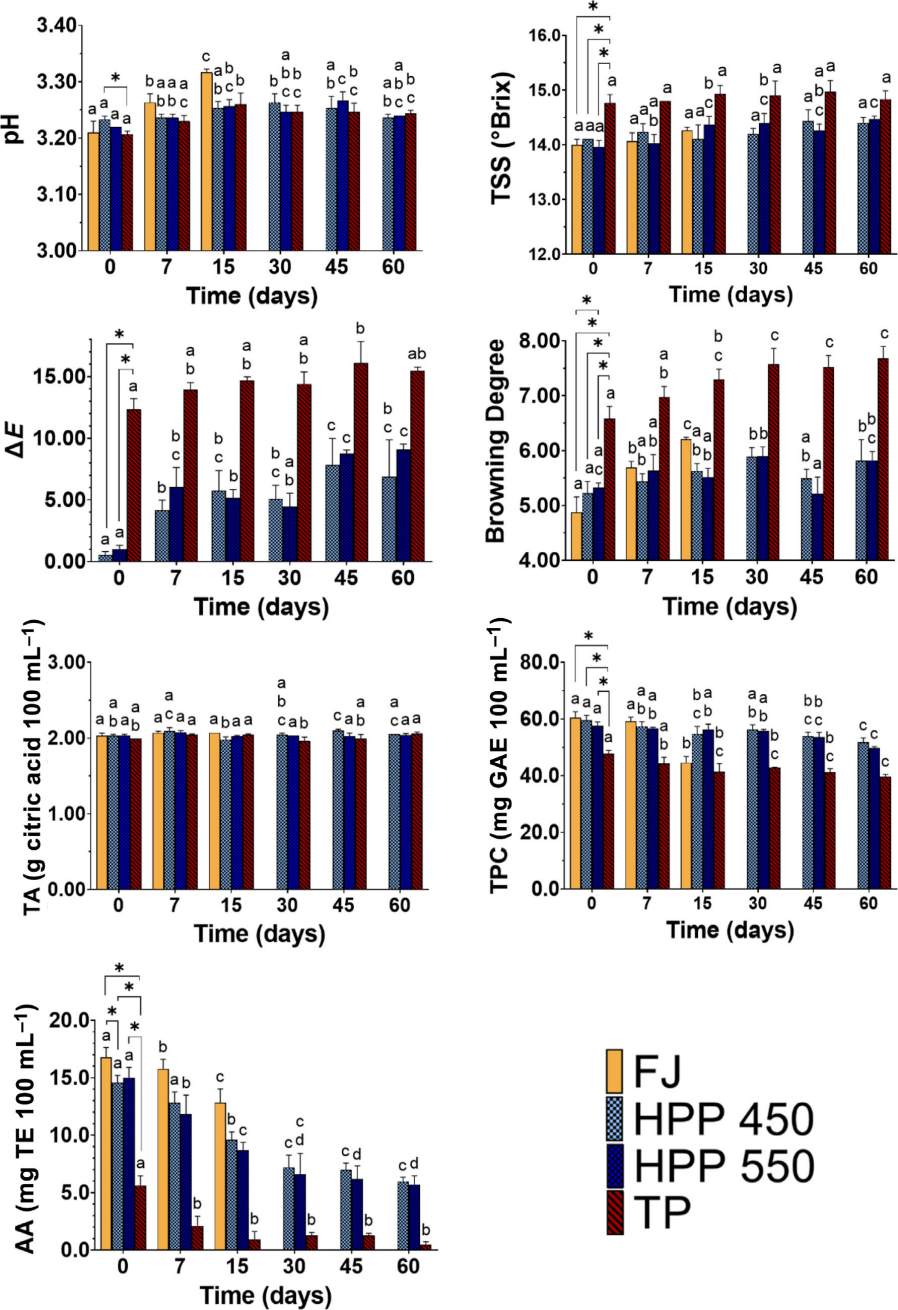
Evolution of physicochemical characteristics on fresh (FJ) and pasteurized passion fruit juices during storage. Physicochemical parameters included pH, total soluble solids (TSS), colour difference (∆*E*), browning degree, titratable acidity (TA), total phenolic compounds (TPC) and antioxidant activity (AA). Pasteurized juices were either high‐pressure pasteurized (at 450 MPa (HPP450) and 550 MPa (HPP550)) or thermically pasteurized (TP). Letters (indicated above the bars) compare the same juice across time, and values with different letters are significantly different (Tukey's test, *P* < 0.05). Comparison between juices was performed at *t* = 0 and statistical different values (Tukey's test, *P* < 0.05) are represented with brackets and asterisks.

The physicochemical evaluation of fresh and pasteurized passion fruit juices during storage under refrigeration conditions is presented in Fig. 4 and includes the determination of pH, BD, Δ*E*, TA, TSS, TPC, and antioxidant activity (AA).

The impact of each pasteurization method on the above‐mentioned parameters can be drawn from the differences observed at *t* = 0 in comparison to FJ. Compared to the initial FJ characteristics, TP has significantly increased TSS and BD, significantly decreased TPC and AA, and caused a significant colour change, as observed in Fig. 4. Although TSS is not usually affected by pasteurization, increases after thermal pasteurization have been observed in some studies and it is usually attributed to water evaporation during heating.^31^, ^32^ Temperature can also promote the hydrolysis‐insoluble polysaccharides (e.g., pectin) into more simple and soluble sugars and also contribute to the TSS increase.^32^, ^33^, ^34^ In fruit juices, the browning is an attribute associated with the occurrence of Maillard reactions as well as enzymatic action of polyphenol oxidase (PPO) and peroxidase (POD).^35^ They are responsible for causing colour changes, off‐flavours and nutrient losses through the formation of melanoidins (in the case of Maillard reactions) or *o*‐quinones that subsequently undergo rapid polymerization, producing pigments that cause fruit browning (in the case of oxidation of phenolic compounds by PPO and POD).^36^, ^37^

Impact on browning and colour is also associated with carotenoid breakdown (0.00288 g kg^−1^ dry weight in purple passion fruit pulp) and vitamin C (1.03 g kg^−1^ dry weight in purple passion fruit pulp) by creating reactive carbonyl groups that act as precursors of non‐enzymatic browning.^23^, ^35^, ^38^ These reactions are probably also contributing to the reduction in AA of TP juice to approximately a third of FJ, which does not match the slighter reduction of TPC. This indicates that, in the case of passion fruit juice, phenolic compounds are probably not the most relevant contributors to AA, but rather other thermosensitive compounds such as carotenoids and vitamin C. Several studies have observed similar impacts of TP on colour, BD, TPC and AA in juices of strawberry, jabuticaba, watermelon and carambola.^39^, ^40^, ^41^, ^42^

On the other hand, HPP, under both conditions tested, resulted in negligible changes in the studied parameters when compared to FJ at *t* = 0, and exhibits a considerably lower impact than TP. In comparison to TP, HPP‐treated juices showed statistically significantly lower values of TSS, Δ*E*, and BD, as well as higher TPC and AA. Several studies have reported similar effects of HPP in better preserving the physicochemical characteristics of fruit juices compared to TP. It has been reported that HPP has no impact on TPC,^39^, ^40^, ^43^ browning, or^39^ AA,^39^, ^43^ and a lower impact on colour change than TP^39^, ^40^, ^44^ in jabuticaba, carambola, and strawberry juices. Neither TP nor HPP treatment impacted pH and TA, since their values were like FJ and were kept stable across storage time. Excluding FJ, which was subject to microbial activity, pH ranged between 3.21 and 3.37 and TA ranged between 1.98 and 2.09 g CA 100 mL^−1^. It has been commonly reported that HPP has an insignificant impact on pH and acidity of juices after processing and through the storage period.^35^, ^41^, ^43^ Despite the values of juice parameters being predominantly altered in TP juices compared with HPP, their tendency over time was similar. TSS evolution remained stable, while BD and Δ*E* had a slight increasing tendency during storage. On the other hand, TPC showed a slight decrease over time (13%, 13% and 17% decrease in HPP450, HPP550, and TP, respectively, at *t* = 60 days), while AA was the most affected characteristic during storage, with a decrease of 59%, 62%, and 92% in HPP450, HPP550, and TP, respectively, after 60 days.

Depending on the matrix, under mild pasteurization conditions PPO and POD may not be completely inactivated, leading to gradual browning over time.^45^, ^46^ Since in the present study mild thermal pasteurization conditions were used, residual activity of PPO and/or POD are probably contributing to the reduction of TPC and subsequent increase of BD and Δ*E*. This can partially contribute to the AA reduction. Typically, PPO is more effectively inactivated by high pressure in acidic environments, which may explain the mitigation of the impacts observed in HPP‐treated juices in comparison to TP.^47^ In addition, non‐enzymatic oxidation can also occur during storage. Even after pasteurization, phenolic compound and carotenoid oxidation can continue due to the presence of residual oxygen or possible metal‐catalysed reactions.^48^, ^49^ Any residual vitamin C (ascorbic acid) not degraded during pasteurization can degrade under exposure to oxygen,^50^ thus reducing its antioxidant capacity.

#### Volatile compound composition

**Figure 5:**
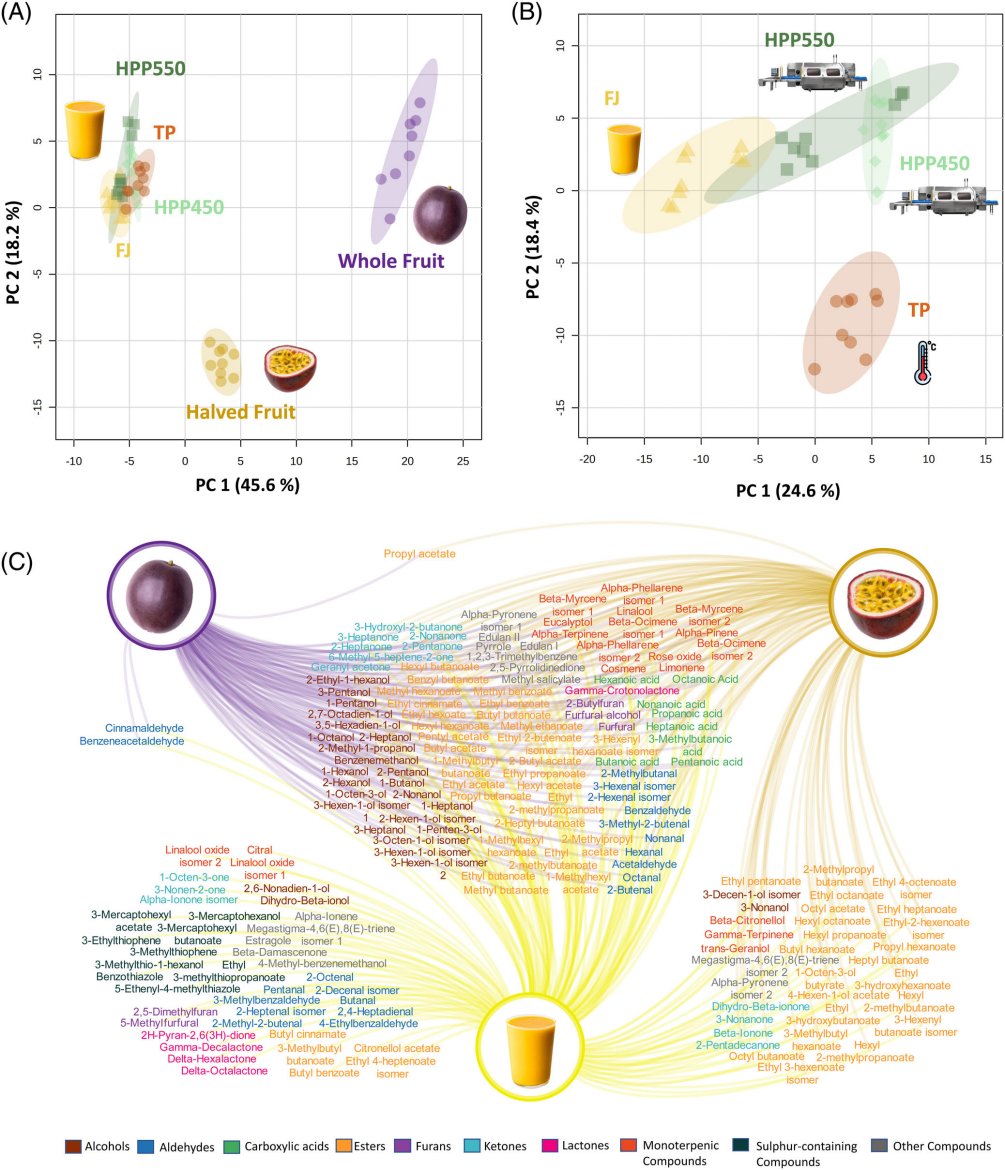
Multivariate and network analysis of volatile compounds in fresh fruit and juice samples. (A) PCA score plot of all the volatile compounds putatively identified in purple passion fruit juice (in fresh juice and after pasteurization: either thermal (TP) or high‐pressure pasteurization at 550 MPa (HPP550) and 450 MPa (HPP450)), halved fruit and whole fruit. Data were normalized by sum and autoscaled. PERMANOVA (permutational multivariate analysis): *F*‐value: 313.96; *R*‐squared: 0.97093; *P*‐value (based on 999 permutations): 0.001. (B) PCA score plot of all the volatile compounds putatively identified in purple passion fruit fresh and pasteurized juice (either TP, HPP550, or HPP450). Data were autoscaled. PERMANOVA: *F*‐value: 66.853; *R*‐squared: 0.8624; *P*‐value (based on 999 permutations): 0.001. (C) Visual representation of the volatile compounds putatively identified in each sample of passion fruit (whole fruit, halved fruit, and juice). Source nodes represent the sample lines connect to compounds detected in each sample. Compounds are colour coded by chemical family.

To evaluate purple passion fruit juice aroma and the potential impact of pasteurization processing, its volatile compound profile was investigated in detail. Compared to the previously analysed samples (WF and HF), juice chromatograms have much more information, with an average of 1841 (FJ) instrumental features obtained (Supporting Information, Table S1). From these, a total of 177 compounds (including alcohols, carboxylic acids, aldehydes, esters, ketones, lactones monoterpenic compounds, sulfur‐containing compounds and furans) were putatively identified and selected for further analysis. This dataset includes 15 of the 19 aroma‐active compounds previously identified in purple passion fruit pulp solvent‐assisted flavour extract.^16^ PCA analysis with the whole dataset of identified compounds shows that, as would be expected, juice samples have a higher similarity to HF than WF (Fig. 5(A)). This is corroborated by the higher number of compounds that are common to these samples (Fig. 5(C)).

Almost all the compounds (99) found in WF were also shared by the remaining samples. It includes, among others, all carboxylic acids, most alcohols and monoterpenic compounds, and approximately half of the esters. When the fruit is cut, there is an increased release of volatile compounds due to the disruption of cellular structures and exposure of the pulp, which intensifies the characteristic aroma of the fruit. These include mainly esters (additional 22 compounds) but also monoterpenic compounds (three) and ketones (four, including β‐ionone, a key aroma compound of the pulp). The juice has an even more complex volatile profile, marked by an increase in the number (as well as in their content) of identified aldehydes and sulfur‐containing compounds, and other compounds often associated with oxidative and thermal degradation reactions that occur during processing (e.g., linalool oxide, 5‐methylfurfural). These results show that the degree of fruit handling directly influences the stability and release of volatile compounds, which may impact the aroma, flavour, and, ultimately, the sensorial quality of the final product.

A PCA analysis with the dataset of all identified compounds in FJ, TP, and HPP juices obtained after processing (Fig. 5(B)) revealed that juice samples separate into different clusters across the principal component space, which indicates that both TP and HPP methods had an impact on the juice volatile composition. PC1 (24.6%) and PC2 (18.4%) explain 43% of the variance in the dataset (which is a moderate representation of the total variability), but the PERMANOVA analysis indicate a high *F*‐value (66.853) and *R*‐squared (0.8624), which supports the clustering observed in the PCA. The *P*‐value (0.001) also indicates that the separation of groups is statistically significant, confirming that the treatments have a significant effect on the volatile compound profile. Among pasteurized juices, HPP samples are closer to the original aroma profile of FJ, evidencing a lower impact of this preservation method, while TP samples form a separate, distant cluster, indicating a more substantial impact on the volatile compound profile.

### Aroma and quality marker evolution throughout refrigerated storage

Pasteurized juices with similar pH to passion fruit (pH = 3.21), stored at 4 °C, can have a shelf life up to 12 weeks.^51^ To evaluate the juice characteristics over such period, a subset of 30 compounds was chosen (Table 1) based on their known sensory relevance and their association with particularly relevant metabolic pathways. This includes the aroma‐active compounds previously identified on purple passion fruit pulp and compounds expected to be formed in pasteurization processing and during the storage period.

**Table 1:** Selected compounds to evaluate the impact of the pasteurization process and storage on volatile characteristics of passion fruit juice

#	Name	Formula	CAS	Inclusion criteria	Reference
1	Ethyl 2‐methylpropanoate	C_6_H_12_O_2_	97‐62‐1	Purple passion fruit aroma‐active compound	16
2	Ethyl butanoate	C_6_H_12_O_2_	105‐54‐4	Purple passion fruit aroma‐active compound	16
3	3‐Hexenal	C_6_H_10_O	6789‐80‐6	Purple passion fruit aroma‐active compound	16
4	Ethyl hexanoate	C_8_H_16_O_2_	123‐66‐0	Purple passion fruit aroma‐active compound	16
5	Geraniol	C^10^H_18_O	106‐24‐1	Purple passion fruit aroma‐active compound	16
6	β‐Ionone	C_13_H_20_O	79‐77‐6	Purple passion fruit aroma‐active compound	16
7	δ‐Octalactone	C_9_H_16_O_2_	104‐61‐0	Purple passion fruit aroma‐active compound	16
8	Ethyl cinnamate	C_11_H_12_O_2_	4610‐69‐9	Purple passion fruit aroma‐active compound	16
9	γ‐Decalactone	C_12_H_22_O_2_	706‐14‐9	Purple passion fruit aroma‐active compound	16
10	Phenylacetaldehyde	C_8_H_8_O	122‐78‐1	Aldehyde formed through Strecker reaction of phenylalanine	52
11	2‐Methylbutanal	C_5_H_10_O	96‐17‐3	Aldehyde formed through Strecker reaction of isoleucine	52
12	Acetaldehyde	C_2_H_4_O	75‐07‐0	Aldehyde formed through Strecker reaction of several amino acids	52
13	Pentanal	C_5_H_10_O	110‐62‐3	Lipid oxidation pathways, (enzymatic and/or non‐enzymatic)	53
14	Hexanal	C_6_H_12_O	66‐25‐1	Lipid oxidation pathways, (enzymatic and/or non‐enzymatic)	53
15	2‐Hexenal	C_6_H_10_O	6728‐26‐3	Lipid oxidation pathways, (enzymatic and/or non‐enzymatic)	53
16	2‐Decenal isomer	C_10_H_18_O	2497‐25‐8	Lipid oxidation pathways, (enzymatic and/or non‐enzymatic)	53
17	β‐Damascenone	C_13_H_18_O	23 696‐85‐7	Carotenoid breakdown, (particularly β‐carotene) pathways, (enzymatic or non‐enzymatic).	54
18	Pyrrole	C_4_H_5_N	109‐97‐7	Maillard reaction product	55
19	5‐Ethenyl‐4‐methylthiazole	C_6_H_7_NS	1759‐28‐0	Maillard reaction product	55
20	Benzothiazole	C_7_H_5_NS	95‐16‐9	Maillard reaction product	55
21	Benzaldehyde	C_7_H_6_O	100‐52‐7	Maillard reaction product	56
22	3‐Methylthiophene	C_5_H_6_S	616‐44‐4	Maillard reaction product	57
23	2‐Methylthiophene	C_5_H_6_S	554‐14‐3	Maillard reaction product	57
24	3‐Ethylthiophene	C_6_H_8_S	1795‐01‐3	Maillard reaction product	58
25	2‐Butylfuran	C_8_H_12_O	4466‐24‐4	Maillard reaction product	59
26	Furfuryl alcohol	C_5_H_6_O_2_	98‐00‐0	Maillard reaction product	60, 61, 62
27	Furfural	C_5_H_4_O_2_	98‐01‐1	Maillard reaction product	60, 61, 62
28	5‐Methylfurfural	C_6_H_6_O_2_	620‐02‐0	Maillard reaction product	60, 61, 62
29	Linalool oxide isomer 1	C_10_H^18^O^2^	5989‐33‐3	Enzymatic or non‐enzymatic oxidation of linalool	21
30	Linalool oxide isomer 2	C_10_H_18_O_2_	5989‐33‐3	Enzymatic or non‐enzymatic oxidation of linalool	21

As extensively documented in the literature, pasteurization methods such as TP and HPP can affect the volatile composition and sensorial quality of fruit juices through a variety of mechanisms, including enzymatic and non‐enzymatic oxidation of several families of compounds. TP, in particular, may induce Maillard reactions, leading not only to the loss of desirable volatiles but also to the formation of off‐flavour compounds.^21^ Among these, furfural and 5‐methylfurfural have been widely recognized as markers of TP and HPP of fruit juices and was therefore included in the set of monitored compounds.^60^, ^61^, ^62^ Other Maillard‐derived volatile compounds, such as pyrrole, thiophenes, thiazoles, and other furanic compounds were also considered due to their relevance in impacting the aroma of food products.^55^, ^56^, ^57^, ^58^, ^59^ Strecker aldehydes, and specifically acetaldehyde (derived from several amino acids), 2‐methylbutanal (from isoleucine), and phenylacetaldehyde (from phenylalanine), were included as indicators of amino acid degradation.^52^ Compounds resulting from oxidation of lipids (e.g., hexanal, 2‐hexenal, 2‐decenal), carotenoid breakdown (e.g., β‐damascenone), and oxidation of monoterpenes (linalool oxides) were also included.^21^, ^53^, ^54^

**Figure 6:**
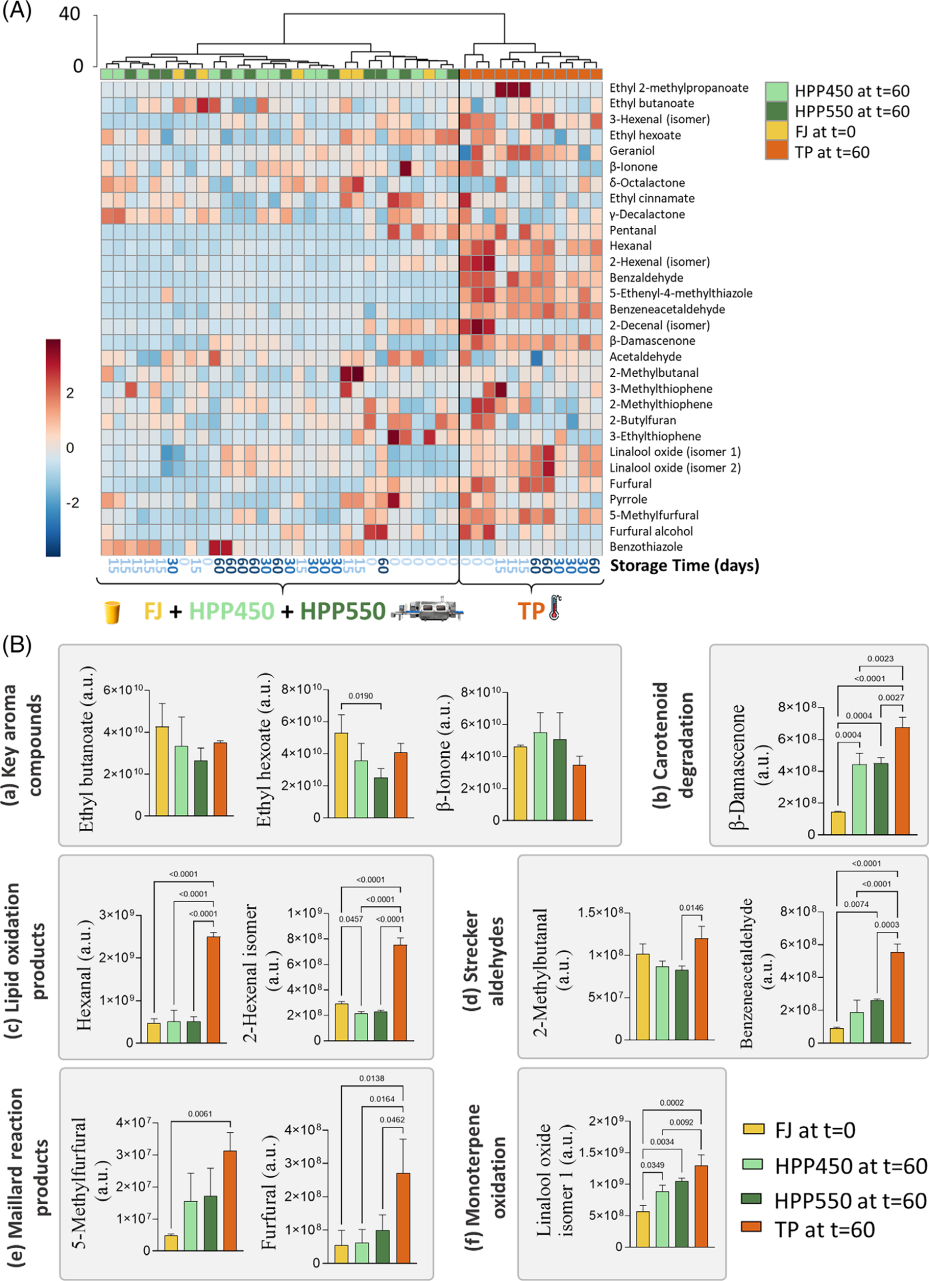
Impact of pasteurization and storage on quality markers in purple passion fruit juice. (A) Hierarchical clustering analysis of the selected volatile compounds (Table 1) in fresh (FJ) and pasteurized (either TP, HPP550, or HPP450) passion fruit juice across storage. Heatmap visualization shows the relative content of each compound, through a chromatic scale (from low (blue) to high chromatographic area (red)), which corresponds to its peak area normalized by autoscaling. (B) Relative content of: (a) key aroma compounds, (b) carotenoid breakdown products, (c) lipid oxidation products, (d) Strecker aldehydes, (e) Maillard reaction products, and (f) monoterpenic oxidation products between unpasteurized fresh juice (FJ) (without storage) and pasteurized (either by TP, HPP450, or HPP550) juice after 60 days of storage. Brackets between bars indicate statistical differences (Tukey's test, *P* < 0.05) of the relative content of the compound between juices; a.u., arbitrary units.

Based on the chromatographic data obtained for these compounds (Supporting Information, Table S2), a heatmap representation of the HCA was constructed (Fig. 6(A)) showing samples organized into two main clusters (Euclidean distance of 40). In one branch of the dendrogram are the TP juices, while the other includes FJ and HPP (at both pressures) juices. This sample clustering according to the similarities of volatile compound content corroborates the previous PCA analysis with all identified compounds (Fig. 5(B)). This shows that juice samples processed by HPP450 and HPP550 are more like FJ than TP, and thus indicate a better preservation of aroma‐related compounds in juices submitted to the HPP pasteurization method. However, the dendrograms also show that besides the processing method, samples also tend to cluster according to storage time, suggesting that both factors significantly influence the evolution of volatile compound composition.

Compared with FJ, the volatile compound content of TP juices after 60 days of storage (Fig. 6(B)) are associated with statistically higher levels of: hexanal and 2‐hexenal (lipid oxidation products); phenylacetaldehyde (aldehyde formed through Strecker reaction of phenylalanine); β‐damascenone (from carotenoid breakdown); furfural and 5‐methylfurfural (Maillard reaction products); and linalool oxide isomers (oxidation products of linalool). HPP juices, on the other hand, only had a significant impact in the increase of phenylacetaldehyde, β‐damascenone and linalool oxide, although in a lower amount than TP. Despite these alterations, no significant changes were observed in key aroma compounds amount in either TP or HPP juices (after 60 days of storage) compared to FJ.

## CONCLUSIONS

The volatilome analysis of WF and HF allowed the identification of 134 and 102 compounds, respectively, 80 of which were detected for the first time in purple passion fruit. The distinctive samples profile was demonstrated, highlighting a clear difference in their volatile compound intensity, chemical family composition, as well as different aroma notes of the compounds that mostly contribute to their distinctiveness (mainly fruity, floral, and balsamic in HF, and earthy, citrus, and fermented odour notes in WF).

Regarding the pasteurization impact on the juice, in comparison to TP, HPP better preserved the physicochemical characteristics of FJ up to 60 days of refrigerated storage in terms of TPC (76.4% *vs*. 59.6%), BD (18.7% *vs*. 56.7% increase), AA (34.8% *vs*. 2.8%), and total colour difference (Δ*E* = 7.99 *vs*. 15.46%). In the case of volatile composition, HPP‐treated juices were also less impactful by having a volatile profile more similar to FJ than TP samples, indicating better preservation of aroma‐related compounds. None of the pasteurized juices had significantly altered levels of passion fruit key aroma compounds after 60 days of storage, but HPP juices exhibited less pronounced chemical changes linked to several reactions (Maillard and Strecker reactions, and carotenoids, lipids, and monoterpenic oxidative reactions).

This work has demonstrated that the aroma‐associated compounds of fresh fruit are substantially released when the fruit is opened. This allows us to infer that the characteristic aroma of passion fruit is released at this stage and is further preserved in the fresh juice, particularly when processed by HPP. Furthermore, the preservation of these aroma‐associated compounds may play a key role in shaping consumer expectations and acceptance. Therefore, future studies involving sensory analysis will be essential to link these chemical data with actual consumer perception, providing a more comprehensive understanding of the sensory drivers of quality. Given the practical implications of our findings, this study will be of significant interest to both food scientists and the juice industry in the development of high‐quality, minimally processed juices that align more closely with consumer expectations.

## CONFLICT OF INTEREST

The authors declare no conflict of interest.

## AUTHOR CONTRIBUTIONS

AMAF performed the experimental work, validation, and formal analysis; wrote the original version, edited the final version, and prepared illustrations. CAP was responsible for HPP experimental work and review of the final version. JAS reviewed the final version. AJDS was responsible for conceptualization and supervision; performed interpretation of the global results, writing, and review and editing of the final version. SMR was responsible for conceptualization, supervision, writing, review and editing of the final version, and funding acquisition. All authors have read and agreed to the published version of the manuscript.

## Supporting information


**Table S1.** Supporting Information.

**Table S2.** Selected compounds with relevant chromatographic data used to determine compounds identification, obtained by HS‐SPME/GC × GC‐ToFMS. These data were used to evaluate the impact of the pasteurization and storage on aroma characteristics of passion fruit juice.

## Data Availability

The data that support the findings of this study are available from the corresponding author upon reasonable request.
